# *S1PR2* variants associated with auditory function in humans and endocochlear potential decline in mouse

**DOI:** 10.1038/srep28964

**Published:** 2016-07-07

**Authors:** Neil J. Ingham, Francesca Carlisle, Selina Pearson, Morag A. Lewis, Annalisa Buniello, Jing Chen, Rivka L. Isaacson, Johanna Pass, Jacqueline K. White, Sally J. Dawson, Karen P. Steel

**Affiliations:** 1 Wellcome Trust Sanger Institute, Wellcome Trust Genome Campus, Hinxton, Cambridge, CB10 1SA, UK; 2 Wolfson Centre for Age-Related Diseases, King’s College London, Guys Campus, London, SE1 1UL, UK; 3 Department of Chemistry, King’s College London, Britannia House, 7 Trinity Street, London, SE1 1DB, UK; 4 UCL Ear Institute, University College London, 332 Gray’s Inn Road, London WC1X 8EE, UK

## Abstract

Progressive hearing loss is very common in the population but we still know little about the underlying pathology. A new spontaneous mouse mutation (stonedeaf, *stdf* ) leading to recessive, early-onset progressive hearing loss was detected and exome sequencing revealed a Thr289Arg substitution in Sphingosine-1-Phosphate Receptor-2 (*S1pr2*). Mutants aged 2 weeks had normal hearing sensitivity, but at 4 weeks most showed variable degrees of hearing impairment, which became severe or profound in all mutants by 14 weeks. Endocochlear potential (EP) was normal at 2 weeks old but was reduced by 4 and 8 weeks old in mutants, and the stria vascularis, which generates the EP, showed degenerative changes. Three independent mouse knockout alleles of *S1pr2* have been described previously, but this is the first time that a reduced EP has been reported. Genomic markers close to the human *S1PR2* gene were significantly associated with auditory thresholds in the 1958 British Birth Cohort (n = 6099), suggesting involvement of S1P signalling in human hearing loss. The finding of early onset loss of EP gives new mechanistic insight into the disease process and suggests that therapies for humans with hearing loss due to S1P signalling defects need to target strial function.

Progressive hearing loss is the most common sensory deficit in the human population and can begin at any age, but we know very little of the underlying molecular or cellular mechanisms involved. Any genes found to be involved in progressive hearing loss can give insight into the molecular pathways and pathological processes leading to deafness. Mouse mutants are valuable tools to provide candidate genes as well as opportunities for detailed mechanistic investigation of the disease process.

Sphingosine-1-phosphate (S1P) signalling is known to be required for normal auditory function from studies of deaf mouse mutants. Mutation of the S1P transporter *Spns2* leads to rapidly-progressive loss of auditory sensitivity^1^ and three independent null mutations of the S1P receptor *S1pr2* have severe elevations in auditory thresholds before 4 weeks old^2^,^3^,^4^. S1P is a lysophospholipid intermediate in the process of degradation of sphingolipids, but it also acts as a signalling molecule with effects both within the cell and outside^5^,^6^. Extracellular S1P signals through five different receptors, S1pr1–5, which in turn activate a variety of intracellular signalling pathways via G-proteins^7^. S1P signalling has been implicated in a range of functions including lymphocyte trafficking^6^,^8^,^9^, macrophage and mast cell function^10^,^11^, angiogenesis^12^, vascular permeability and tone^13^ and bone remodelling^14^. It is not clear which if any of these functions is involved in normal hearing.

Here we report a missense mutation in *S1pr2* that arose spontaneously in mice generated by a large scale targeted mutagenesis program^15^,^16^. We describe the identification of the causative mutation in the *S1pr2* gene by linkage analysis and exome sequencing, the rapidly progressive hearing loss associated with a decline in the endocochlear potential, and subsequent loss of cochlear hair cells. Furthermore, we found that genomic variants close to the human *S1PR2* gene were significantly associated with auditory thresholds in a large population sample, the 1958 British Birth Cohort. The new mouse mutant we report here provides mechanistic insight into the pathological processes underlying S1PR2-related hearing loss.

## Results

As part of the Sanger Institute Mouse Genetics Project, new lines of mutant mice on a C57BL/6N genetic background are screened at 14 weeks old using an electrophysiological test of hearing, the Auditory Brainstem Response (ABR)^16^,^17^. In one line carrying a targeted disruption of the *Mms22l* gene (MMS22-like, DNA repair protein), most mice had normal ABR thresholds (n = 7), but others (n = 4) showed no response to sound stimuli, including one wildtype littermate used as a control. The deafness phenotype was isolated in a new mouse colony and showed transmission consistent with monogenic autosomal recessive inheritance with full penetrance. We named the mutant allele stonedeaf (*stdf* ).

**Figure 1:**
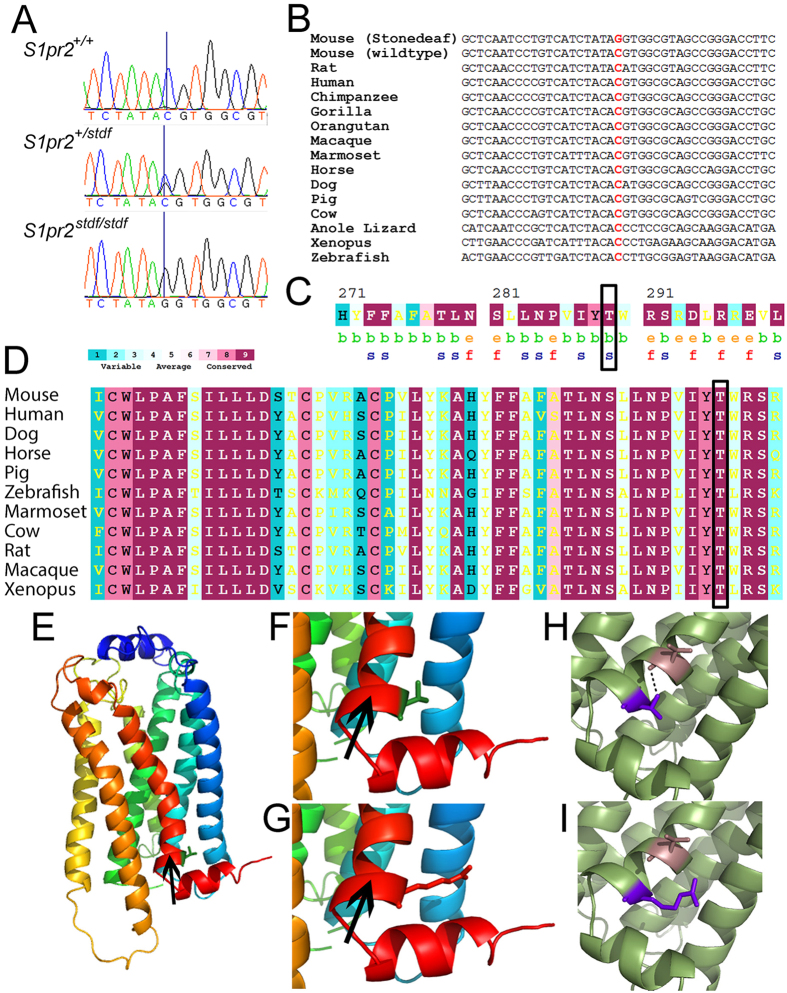
The stonedeaf mutation is a G > C transition in *S1pr2* leading to a threonine to arginine substitution. (**A**) Trace showing the G > C SNV in wildtype (top), heterozygote (middle) and homozygote mutant (bottom) mice. (**B**) The base at 9:20772109 (indicated in red) is conserved between mouse, human, xenopus and zebrafish. Note that S1pr2 is on the reverse strand, and so the G > C SNV appears as C > G when the sequence is oriented in the direction of reading of the gene, as in **A** and **B** in this figure. (**C**) ConSurf analysis of the residues; 289T is predicted to be buried (b) and highly conserved, and thus likely to be a structural residue (s). (**D**) ConSurf-annotated clustal of S1pr2 protein sequences. 289T is highly conserved. In (**C**,**D**), yellow lettering indicates insufficient data for a reliable calculation of conservation. (**E**) Structural model of the wildtype S1pr2 protein based on Phyre2 modelling from the S1pr1 structure. The seven transmembrane helices are shown in different colours. A black arrow points to the snorkelling helix (coloured red) referred to in the text. (**F**) A zoomed view of the wildtype 289T residue (green stick form) (**G**). A zoomed view of the Arginine residue (red stick form) in the T289R substitution of *S1pr2*^*stdf*^. The effect of the mutation on the S1pr2 hydrogen bond network (based on Phyre2 modelling) is shown in **H** and **I**. (**H**) In the wildtype, T289 (purple) forms a single hydrogen bond (dotted line) with V286 (brown). (**I**) In the *S1pr2*^*stdf*^ mutation, R289 (purple) can no longer form a hydrogen bond with V286 (brown) or any other residues.

The *stdf* mutation was mapped to a 10 Mbp interval of proximal chromosome 9 by linkage analysis of offspring from a [(*stdf*/*stdf* x C3HeB/FeJ)F1 x *stdf*/*stdf* ] backcross. DNA from two distantly-related affected mice was submitted for exome sequencing and the data filtered and analysed (Tables 1, 2, 3, 4, 5). A large number of small structural variations were detected by Pindel across the whole genome. After filtering, 4 remained in the critical 10 Mbp region, but they were not located in coding regions, and were not confirmed by capillary sequencing. Two were present in the ancestral ES cell line (Table 3). Single nucleotide variants (SNVs) called by SAMtools were filtered as described (Table 4). Only 7 were within the critical 10 Mbp region (Tables 4 and 5). Of those 7 SNVs, only three were in coding regions; one was synonymous (in *Rdh8*) and the other two resulted in amino acid changes. One of the two nonsynonymous SNVs (in *Bmper*) was found in the ancestral ES cell line used in the creation of the knockout from which the stonedeaf allele arose (Table 5) and the SNV (A > C substitution producing an amino acid change of T570P) was predicted to be neutral, so we considered this to be an unlikely candidate. The remaining SNV (a G > C transition in *S1pr2*; Fig. 1A), resulted in a threonine to arginine change (T289R), which was predicted by both NetDiseaseSNP^18^ and PhD-SNP^19^,^20^ to be deleterious. The affected threonine residue is highly conserved (Fig. 1B,D), located in a transmembrane domain, and was predicted by ConSurf 21,22 to be structural (Fig. 1C). Genotyping a large population (n = 204) of mice from the stonedeaf colony for this mutation by capillary sequencing supported its causative nature; all mice with raised thresholds (>4 weeks old) were homozygous for the mutation, whereas mice with normal thresholds were either *S1pr2*^+/*stdf*^ or *S1pr2*^+/+^.

S1pr2 was subjected to protein structure prediction using Phyre2^23^, based on the X-Ray crystal structure of homologous receptor, S1pr1^24^ and the T289R mutation was introduced using Pymol™ (DeLano Scientific LLC) (Fig. 1E–I). The mutation sits within a hydrophobic pocket and is likely to cause some local disruption including changing available hydrogen bonds (Fig. 1H,I). The mutation site (289T) is situated on the boundary between the membrane and intracellular region of the receptor. It might be anticipated that the replacement of threonine at position 289 with an arginine residue would have little effect on the protein’s position within the membrane. However, membrane proteins are often highly sensitive to mutation and modest differences in the tilt angle of the transmembrane region relative to the membrane surface, or slight changes in insertion depth have been known to cause significant effects on function (several such cases have been reviewed^25^). In S1pr2, the position of the snorkelling helix just above the aberrant arginine residue may be affected which might perturb intracellular signalling function (See Fig. 1E–G). The structural modelling therefore supports our conclusion that the T289R mutation is the *stdf* causal mutation.

**Figure 2:**
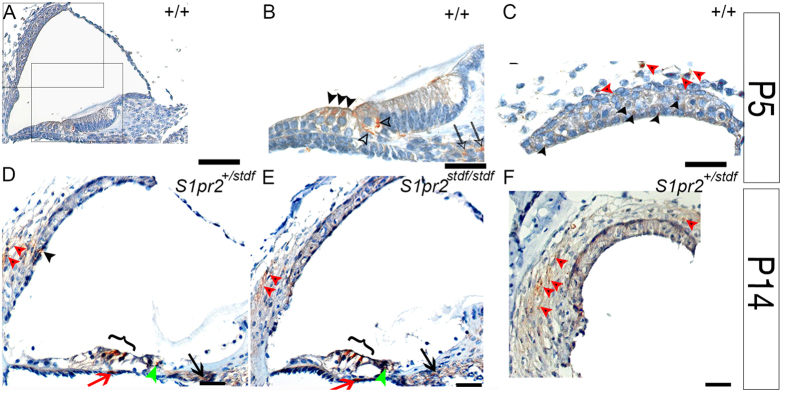
Immunohistochemical localisation of S1pr2. (**A**–**C**) Expression pattern of S1pr2 in the wildtype cochlea at P5 (brown staining indicates S1pr2 labelling). S1pr2 is mainly expressed in scattered cells of the stria vascularis (black arrowheads in **C**), the developing spiral ligament (red arrowheads in **C**), Outer Hair Cells (OHCs, black arrowheads in **B**), cells of the spiral ganglion (arrows in **B**) and spiral ganglion neuron terminals innervating the Inner Hair Cells (IHCs, open arrowheads in **B**). (**D**,**E**) S1pr2 cochlear expression at P14 in stonedeaf mutant (*S1pr2*^*stdf/stdf*^ ) and littermate controls (*S1pr2*^+/*stdf*^ ). We detect expression of S1pr2 mainly in spiral ligament fibrocytes (red arrowheads), OHCs (bracket), individual cells in the stria vascularis (black arrowhead), basilar membrane (red arrows) spiral ganglion cells (arrows) and spiral ganglion neuron terminals below the IHCs (green arrowheads). We did not detect a difference in S1pr2 expression distribution, or any obvious difference in labelling intensity, between stonedeaf mutants and littermate controls. F: Expanded view of S1pr2 expression in the spiral ligament fibrocytes (red arrowheads). Scale Bars: (**A**,**D**–**F**) 20 μm. (**B**,**C**) 10 μm.

Immunohistochemistry was used at postnatal day (P)5 and P14 (Fig. 2) to reveal expression of S1pr2 mainly in the cell bodies of inner and outer hair cells (Fig. 2C,D), and in the stria vascularis and spiral ligament fibrocytes which are important for the recycling of ions into the scala media (Fig. 2B,F), and in spiral ganglion cells. The same pattern of expression was observed at P28 (data not shown). S1pr2 expression was detected with a similar distribution in the *S1pr2*^*stdf*/*stdf*^ mutants (Fig. 2E), which is not surprising as the point mutation is unlikely to stop protein translation.

**Figure 3:**
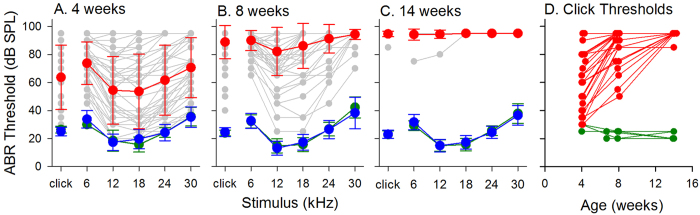
Auditory Brainstem Response (ABR) thresholds in *S1pr2*^*stdf*^ mice. (**A**–**C**) Mean ABR thresholds (±SD) recorded in *S1pr2*^+/+^ (green), *S1pr2*^+/*stdf*^ (blue) and *S1pr2*^*stdf*/*stdf*^ (red) mice aged 4 weeks old (A, *S1pr2*^+/+^ n = 7, *S1pr2*^+/*stdf*^ n = 34, *S1pr2*^*stdf*/*stdf*^ n = 44), 8 weeks old (B, *S1pr2*^+/+^ n = 20, *S1pr2*^+/*stdf*^ n = 75, *S1pr2*^*stdf*/*stdf*^ n = 71) and 14 weeks old (C, *S1pr2*^+/+^ n = 7, *S1pr2*^+/*stdf*^ n = 26, *S1pr2*^*stdf*/*stdf*^ n = 25). Individual homozygous mouse thresholds are plotted as grey symbols and lines to emphasise the scatter of the data. (**D**) Click thresholds recorded longitudinally in *S1pr2*^+/+^ (green, n = 7), and *S1pr2*^*stdf*/*stdf*^ (red, n = 28) mice are plotted as a function of age (from 4–14 weeks).

Changes in auditory sensitivity with age were assessed by ABRs^17^, using capillary sequencing to assign genotype (Fig. 3A–C). Mice homozygous for the stonedeaf mutation demonstrated a wide range of auditory sensitivity at 4 weeks old (Fig. 3A), while thresholds were higher at 8 and 14 weeks old (Fig. 3A–C). For some of these mice (n = 62), ABR measurements were made longitudinally at 4, 8 and 14 weeks old (Fig. 3D). A progressive deterioration in threshold with increasing age was apparent in *S1pr2*^*stdf*/*stdf*^ mice (n = 28). Wildtype (n = 7) and heterozygous (n = 27, not shown) mice maintained good sensitivity of threshold as age increased.

**Figure 4:**
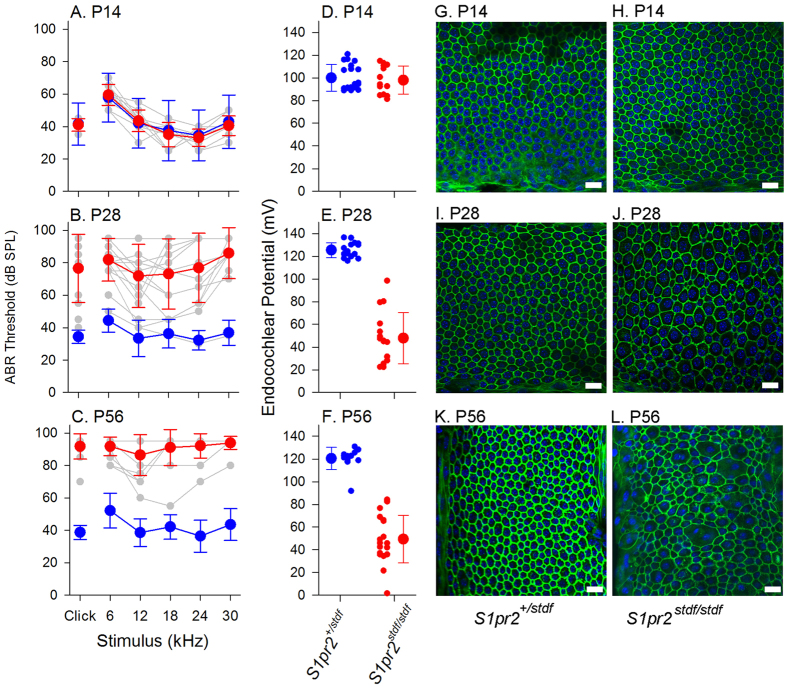
Auditory Brainstem Responses (ABR), Endocochlear Potential (EP) and strial structure in stonedeaf mice. Left column: Mean ABR thresholds are plotted for mice aged P14 (**A**), P28 (**B**) and P56 (**C**), for control *S1pr2*^+/*stdf*^ mice (blue) and for mutant *S1pr2*^*stdf*/*stdf*^ (red). Individual *S1pr2*^*stdf*/*stdf*^ thresholds are given shown as grey symbols and lines , to illustrate the range of data. Middle column: EP measurements with mean ±SD. Right columns: Whole mounts of middle turn stria vascularis with filamentous actin (green) and marginal cell nuclei (blue); scale bar = 20 μm. (**A**) In P14 mice, ABR thresholds of *S1pr2*^*stdf*/*stdf*^ mice (red) (n = 6) were comparable to those recorded in *S1pr2*^+/*stdf*^ littermate controls (blue) (n = 10). (**D**) P14 EPs recorded were also of comparable magnitude in *S1pr2*^*stdf/stdf*^ mice (n = 13; mean EP ± SD = 98.0 ± 12.5 mV; range 81.3–114.9 mV) and *S1pr2*^+*/stdf*^ littermate controls (n = 20; mean EP ± SD = 100.0 ± 11.8mV; range 88.9–120.9 mV). (**G**,**H**) P14 strial marginal cell boundaries had a similar appearance in *S1pr2*^+/*stdf*^ controls and in *S1pr2*^*stdf*/*stdf*^ mice. (**B**) At P28, ABR thresholds of *S1pr2*^+/*stdf*^ control mice were maturing towards adult levels (n = 16), but those of *S1pr2*^*stdf*/*stdf*^ mutants (n = 16) demonstrated a wide range of sensitivity. (**E**) At P28, EP in *S1pr2*^+/*stdf*^ control mice had reached fully mature levels (n = 16; 125.5 ± 6.5 mV, range 116.0–136.6 mV) but *S1pr2*^*stdf*/*stdf*^ mutants demonstrated a reduced and variable EP (n = 16; 47.9 ± 22.2 mV, range 22.4–98.5 mV). (**I**,**J**) Strial marginal cell boundaries in P28 *S1pr2*^*stdf*/*stdf*^ mice started to become less regular (panel J) compared to those of *S1pr2*^+/*stdf*^ controls (panel I). (**C**) At P56, ABR thresholds of *S1pr2*^*stdf*/*stdf*^ mutants (n = 14) showed further elevations compared to littermate controls (n = 7). (**F**) Mean EP from P56 mutant mice remained low (n = 20; 53.7 ± 17.9 mV, range 1.6–84.2 mV) while *S1pr2*^+/*stdf*^ control mice maintained normal EPs (n = 12; 120.5 ± 9.8 mV, range 91.8–130.9 mV). (**K**,**L**) Strial marginal cell boundaries became more irregular at P56 in mutants.

Expression of S1pr2 in the lateral wall of the cochlear duct suggested a potential role for S1P signalling in generation of the high resting potential, the endocochlear potential (EP), in the endolymph bathing the upper surface of the sensory hair cells. EP is essential for normal hair cell function, providing a strong driving force across transduction channels. We measured EP in mice aged P14, P28 and P56, in some cases following ABR recording. In P14 mice, ABR thresholds of *S1pr2*^*stdf*/*stdf*^ mice were comparable to those recorded in *S1pr2*^+/*stdf*^ littermate controls (Fig. 4A). EPs recorded were also of comparable magnitude in *S1pr2*^*stdf*/*stdf*^ mice (98.0 ± 12.5 mV) and *S1pr2*^+/*stdf*^ littermate controls (100.0 ± 11.8 mV) (Fig. 4D). At P28, ABR thresholds of control mice were maturing towards adult levels, but homozygous mutants demonstrated a wide range of sensitivity (Fig. 4B). At P28, EP in control (heterozygote) mice had reached fully mature levels (125.5 ± 6.5 mV) but mutants demonstrated a reduced and variable EP (47.9 ± 22.2 mV) (Fig. 4E). At P56, ABR thresholds of mutants showed further elevations compared to littermate controls (Fig. 4C) and EP remained low (53.7 ± 17.9 mV). Littermate control heterozygotes maintained normal EPs at P56 (120.5 ± 9.8 mV) (Fig. 4F). In summary, at early stages of the pathological process, ABR thresholds increase in line with the reduction in EP.

**Figure 5:**
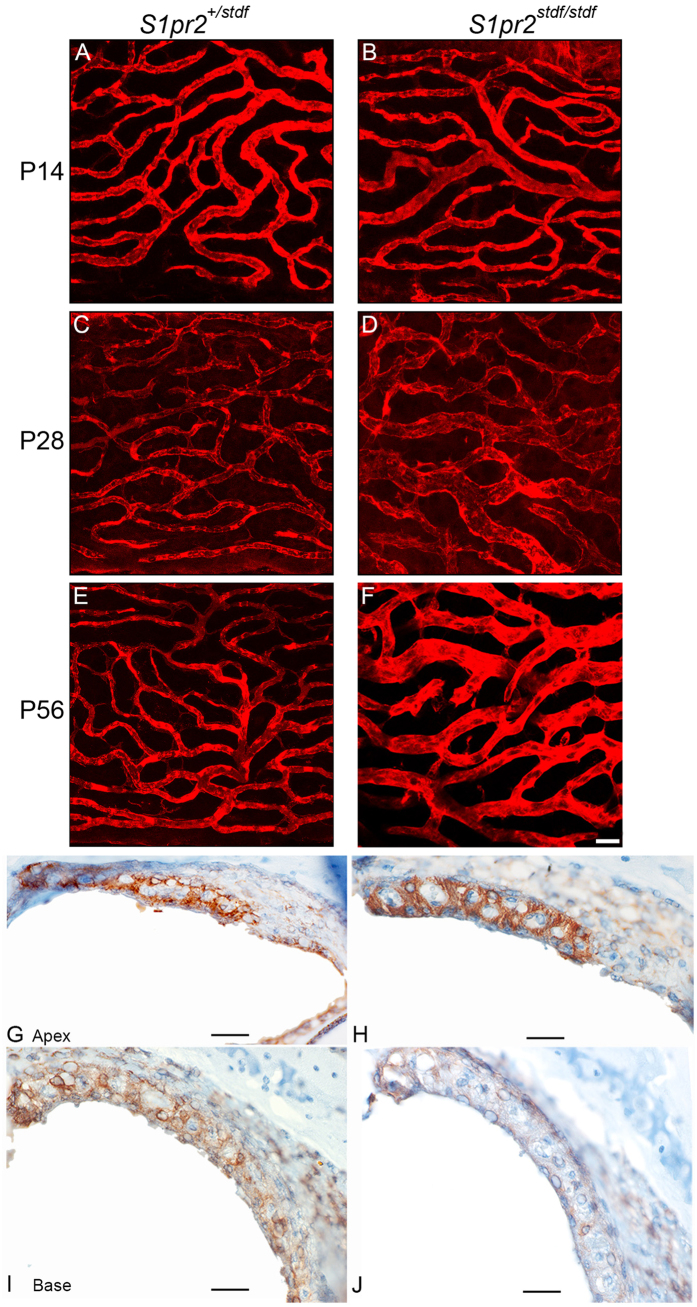
Capillary expansion and Kcnj10 expression in the stria vascularis of *S1pr2*^*stdf*^ mice. Capillaries in the middle turn stria vascularis of mutants, shown for *S1pr2*^*stdf/stdf*^ mutants (**B**,**D**,**F**) and in *S1pr2*^+/+^ littermate controls (**A,C,E)** at P14 (**A,B**), P28 (**C,D**) and P56 (**E,F**). Capillaries appeared swollen at P28 and P56 in *S1pr2*^*stdf/stdf*^ mutants (**D**) compared to *S1pr2*^+/+^ littermate controls (**C**). Panels **A**–**F** were imaged at the same magnification; scale bar (in **F**): 20 μm. Kcnj10 (brown label) was expressed at P28 in the stria vascularis intermediate cells in both *S1pr2*^*stdf/stdf*^ mutants (**H,J**) and in *S1pr2*^+/+^ littermate controls (**G,I**), in the apical and basal turns of the cochlea. Labelling appeared strongest towards the apex (**G,H**) and less strong towards the base (**I,J**). Scale Bars (**G–J**): 10 μm. Sections of the entire inner ear were examined, n = 3 mice for each genotype.

The stria vascularis on the lateral wall of the cochlear duct is the major site of EP generation, through active pumping of K^+^ ions into the endolymph. As EP was reduced in mutants, we examined the stria vascularis in whole mount preparations labelled with isolectin GS-IB4 to highlight capillaries and phalloidin to indicate filamentous actin at the boundaries of the marginal cells lining the luminal surface of the stria^1^. We examined strial samples from mice aged P14, P28 and P56. Strial marginal cell boundaries appeared normal in stonedeaf homozygotes at P14 (Fig. 4G,H). However, at P28, these boundaries were less regular in mutants than in heterozygous controls, showing a mixture of larger and smaller boundaries, typically losing their normal hexagonal/pentagonal shapes (Fig. 4I,J). These changes were seen intermittently with some normal segments in between. This structural defect progressed with age. At P56, some extremely large or small marginal cell boundaries were seen (Fig. 4K,L). Strial capillaries displayed clear dilation in patches in some of the mutants, which was not seen in any of the controls (Fig. 5A–F).

Kcnj10 is an ATP-sensitive inward rectifying K^+^ channel which plays a crucial role in the movement of K^+^ into the endolymph to establish the high [K^+^] and positive EP^26^, so we looked for expression in the stria vascularis of stonedeaf mutants at P5 and P28. Expression appeared as strong in mutants as in controls at P5 but by P28, immunolabelling was weaker in the basal turn of mutants compared with controls (Fig. 5G–J).

**Figure 6:**
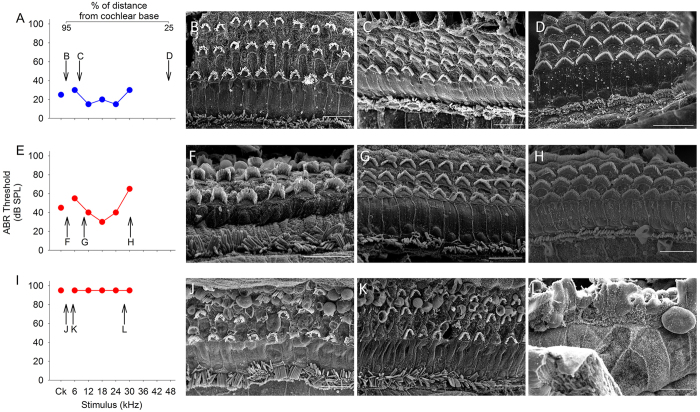
ABR data from individual mice with corresponding scanning electron micrographs. ABR thresholds from one *S1pr2*^+/*stdf*^ mouse (**A**) and two *S1pr2*^*stdf/stdf*^ mice (**E,I**) recorded at four weeks with scanning electron micrographs from the same mice at five weeks old. (**B–D**) show the apical, middle and basal turns respectively of the *S1pr2*^+*/stdf*^ mouse; the corresponding best frequency for each region illustrated is indicated on the audiogram (**A**). (**F–H**) likewise show the apical, middle and basal turns of the *S1pr2*^*stdf/stdf*^ mouse whose audiogram is shown in (**E**), with the corresponding frequencies marked. Although this mouse has raised thresholds, the organ of Corti appears normal. (**J–L**) show the apical, middle and basal turns of a *S1pr2*^*stdf/stdf*^ mouse with no response to stimuli at all (**I**). No hair cells are visible in the basal turn (**L**), but some remain in the mid-apical (**K**) and apical (**J**) turns. Scale bar = 10 μm.

No gross anatomical defects were found in the middle ear, ossicles or inner ear in stonedeaf mutants. The organ of Corti was then examined by scanning electron microscopy. All mice were ABR-tested one week before fixation and later genotyped by sequencing. In five-week-old mutants, some abnormalities of the organ of Corti were noted only in severely impaired *S1pr2*^*stdf*/*stdf*^ mice. Homozygotes with click thresholds of 95dB SPL, or with no ABRs at all, showed OHC degeneration from the middle turn of the cochlea towards the base, while homozygotes with click thresholds at or better than 80dB SPL appeared to have largely normal organs of Corti (Fig. 6). Degeneration of hair cells was much more extensive in 9-week-old affected mice. Only the extreme apical 10–20% of the organ of Corti retained some inner and outer hair cells in *S1pr2*^*stdf*/*stdf*^ mice at this age and there was extensive degeneration in more basal regions (data not shown). A base-to-apex gradient in hair cell degeneration is a common pathological pattern.

We next asked if the targeted *Mms22l* mutation had any impact upon the auditory phenotype. Following identification of the *stdf* mutation, mice with ABR data were divided according to both the *S1pr2* genotype and the *Mms22l* genotype (n = 73 mice aged 4 weeks, 118 mice aged 8–10 weeks, 14 mice aged 44 weeks). However, the presence or absence of the targeted *Mms22l* allele did not significantly affect ABR thresholds within any *S1pr2* genotype and age group (Mann-Whitney Rank Sum Test, p > 0.05 in all cases).

**Figure 7:**
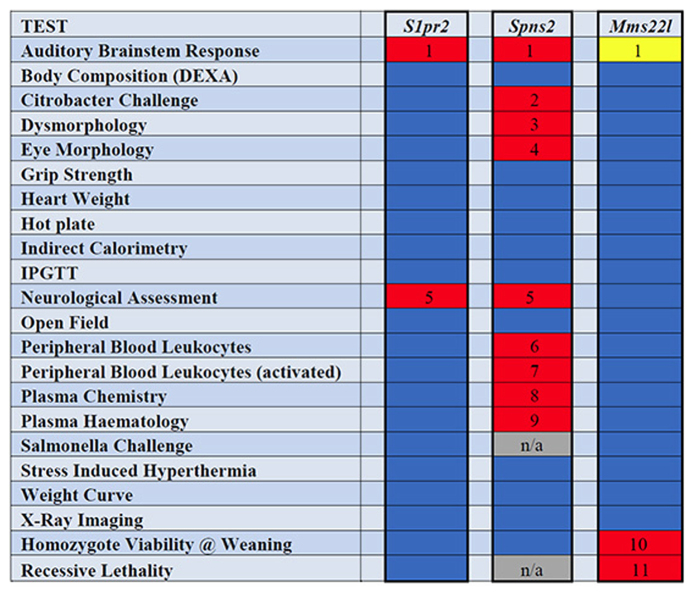
A heatmap to indicate significant phenotyping parameters obtained for the spontaneous mutant line *S1pr2* and 2 targeted mutant lines, *Spns2* and *Mms22l*. Red cells indicate which of 22 phenotyping assays had at least one parameter that was significantly affected compared to controls. Blue cells indicate no difference to controls. The yellow cell for Mms22l indicates the assay (ABR) where results did not segregate with the targeted genotype and led to the identification of the stonedeaf mutation. The red cell indicating abnormal neurological features results from a lack of startle response or Preyer reflex, the ear flick response to sound, which is part of the neurological assessment; all other neurological tests gave normal responses. The phenotypic differences noted are numbered as follows: 1. Elevated ABR thresholds, 2. Abnormal Bacterial Shedding, 3. Abnormal eye coloration, 4. Abnormal corneal opacity, corneal vascularisation, eyelid closure, iris pupil shape, 5. Abnormal startle response, 6. Abnormal % B Cell CD19+, % Granulocyte Gr1+, % Mature B Cell IgD+, % Monocyte, % NK Cell, % NKT Cell, % T Cell CD3+, % T Cell CD4+, % T Cell CD8+, 7. Abnormal % B Cell CD19+, % Granulocyte Gr1+, % Mature B Cell IgD+, % Monocyte, % T Cell CD3+, % T Cell CD4+, % T Cell CD8+, 8. Abnormal glucose, total bilirubin, 9. Abnormal white blood cell count, 10. Abnormal viability at weaning, 11. Homozygotes show recessive lethality. (Details of these phenotyping platforms can be found in ref. 16).

To assess the possibility that other phenotypes were associated with the *S1pr2*^*stdf*^ mutation, we obtained DNA samples from 39 mice from the *Mms22l* line that had been screened as part of the Mouse Genetics Project^16^. These samples were genotyped for the *S1pr2*^*stdf*^ mutation and mice grouped into new cohorts that were wildtype (n = 10), heterozygous (n = 17) and homozygous (n = 12) for the *S1pr2*^*stdf*^ mutation. Phenotyping data for 252 parameters measured across 22 broad assay groups^16^ were obtained for these 39 mice and analysed to determine significant phenotypes. A summary of these data are shown in Fig. 7 as a heatmap, with data for the stonedeaf mutants alongside that of the *Mms22l* mutants and of *Spns2*, another mutant line affecting an S1P transporter protein involved in the same signalling pathway and with a similar auditory phenotype^1^. The *S1pr2*^*stdf*^ mutation had a selective effect only on hearing, and showed no other abnormalities over the range of phenotyping tests (the red box in Fig. 7 indicating abnormal neurological features results from a lack of Preyer reflex, the ear flick response to sound, which is part of the neurological assessment). The targeted *Mms22l* allele led to lethality in homozygotes, so heterozygotes were screened but no other anomalies were detected other than the three individuals that were deaf and later proved to be homozygous for the stonedeaf mutation. In contrast, the *Spns2* mutants showed deafness and a number of additional abnormal phenotypes (Fig. 7), including eye defects^1^ and various anomalies of the immune system, especially associated with leucocytes^9^. S1P signalling effects on the immune system and on retinal angiogenesis are mediated through the *S1pr1* gene (for example^27^,^28^,^29^,^30^) and similar deficiencies would not be expected in the *S1pr2*^*stdf*^ mutant mouse.

Finally, we investigated the potential involvement of *S1PR2* in human hearing loss by asking if variants in this gene show association with auditory thresholds in the 1958 British Birth Cohort^31^. Hearing thresholds measured at 1 and 4 kHz at the age of 44–45 and genotype data imputed to the 1000 Genomes dataset (March 2012 release) were used in this association study. Both datasets were available for 6,099 individuals. SNPs within 0.1 Mb of the *S1PR2* gene as well as within the gene itself were interrogated as a candidate gene association. For 1 kHz audiometric thresholds, the adjacent SNP rs74930654 (which is 27 kb from the end of *S1PR2*, on the reverse strand) showed the most significant association, with a p value of 0.001644. For 4 kHz thresholds, the most significant association was with rs201930568 (located 100 kb before the start of *S1PR2*, on the reverse strand), with a p value 0.001105. These findings suggest that variants affecting the *S1PR2* gene do contribute to auditory thresholds in the UK population.

## Discussion

Stonedeaf is a new missense mutation of *S1pr2* with early-onset, progressive hearing loss with no other phenotypes apparent besides the hearing defect. The earliest detected changes in the cochlea were the reduced EP measurements and morphological changes of the stria vascularis. The appearance of hair cell degeneration in stonedeaf mice appeared to be a secondary phenomenon, occurring after the onset of pathological changes to the stria vascularis, EP and ABR thresholds. Indeed, normal-looking hair cells were seen in the apex of the cochlea in 5- and 9-week-old mice with no measureable ABRs. Secondary hair cell degeneration resulting from a reduced EP has been described before in mouse mutants where (unlike *S1pr2*) there is no expression of the mutant gene in the hair cells, such as in mice with mutations of the *Kit* gene^32^. A normal EP level may be critical for the maintenance of the sensory hair cells, which are specialised to survive in an environment where their upper surface is bathed in endolymph, so any change in voltage or ionic composition of endolymph may disrupt their homeostasis. *S1pr2* is expressed in hair cells so we cannot rule out a direct effect of the mutation on hair cells, but as the earliest cochlear defect we saw was the reduction in EP, this is likely to be the primary pathological event leading to raised ABR thresholds and secondary hair cell degeneration.

The changes we observed in the strial blood vessels may reflect a primary change due to abnormal S1P signalling, which has previously been reported to have an impact on vascular tone of the modiolar artery^33^. Alternatively, the dilation we see may be a consequence of abnormal strial function, leading to both reduced EP and compensatory changes of the vasculature in response to local metabolic demand. Previous reports of mutants with strial dysfunction due to a lack of intermediate cells suggest that the strial vasculature responds to metabolic demand leading to small capillaries where the stria is not functional^34^.

The pattern of reduced EP and altered strial morphology early in the progression of the hearing loss is very similar to that seen in mice with a mutation of *Spns2*, another component of S1P signalling^1^. A progression to fewer, larger marginal cells has been described previously in ageing BALBc and CBA/CaJ mice as their EP becomes lower^35^,^36^ and in other mutant mice with EP defects such as the pendrin mutant^37^. Three independent knockout alleles of *S1pr2* have been reported previously^2^,^3^,^4^, and disrupted marginal cell boundaries and expanded strial capillaries were described in one of these mutant lines^3^ although no EP measurements were made. These knockout mice all showed more severe hearing impairment than we see in the stonedeaf mutant, although none of them reported thresholds as early as P14 so it is not clear if there was normal development followed by very rapid loss of sensitivity. We find thresholds are normal in *S1pr2*^*stdf*^ homozygotes at P14, and by P28 there is considerable variability in thresholds despite all the mice having the same genetic background (C57BL/6N) and being housed in identical environmental conditions. In two targeted null alleles of *S1pr2*, severe hearing impairment was reported in all mice tested at P22^2^ or 4 weeks old^3^, so the stonedeaf missense mutation may produce a hypomorphic effect on S1pr2 function and slower progression of the phenotype. The three null alleles of *S1pr2* were also reported to show severely abnormal stria vascularis morphology, vestibular defects and progressive spiral ganglion loss. We did not see any indications of vestibular defects in *S1pr2*^*stdf*^ homozygotes, but we did find stria vascularis structural defects that progressed with age and were similar to those previously reported in a null allele^3^.

In conclusion, we report here for the first time that EP is reduced resulting from an *S1pr2* mutation, a feature that was not reported in the three knockout *S1pr2* mutants. Furthermore, we found that the *S1pr2*^*stdf*^ homozygotes show normal thresholds and EP around the onset of hearing, progressing over the following few weeks to severe or profound deafness, greatly reduced EP and presumed secondary hair cell degeneration. The delayed loss of auditory function seen in *S1pr2*^*stdf*^ homozygotes suggests that the functional processes mediated through S1P-signalling are not critical for the development of hearing, but instead are required for the ongoing maintenance of cochlear function once hearing has been established. This suggests the possibility of therapeutic intervention to prevent the hearing loss if diagnosed early in the pathological process. This is particularly relevant given the recent discovery of missense mutations in human *S1PR2* 38 and our discovery of significant association between genomic markers close to the *S1PR2* gene and auditory thresholds in the human population. These findings emphasize the need for accurate diagnosis and development of treatments to restore EP in such cases where S1P signalling is implicated; simply attempting to regenerate hair cells, for example, would not restore hearing if a patient has a pathology involving EP decline. Discussion and research on developing treatments for hearing loss often focus on sensory hair cells, but our report provides the first clear example of the involvement of S1PR2 in EP generation and the need to restore EP if treatments are to be successful.

## Materials and Methods

### Ethics statement

Mouse studies were carried out in accordance with UK Home Office regulations and the UK Animals (Scientific Procedures) Act of 1986 (ASPA) under UK Home Office licences, and the study was approved by the King’s College London Ethical Review Committees. Mice were culled using methods approved under these licences to minimize any possibility of suffering.

### Origin and identification of mutant allele

The stonedeaf (*stdf* ) mutation arose in mice derived from the Sanger Institute Mouse Genetics Project, which uses targeted ES cells to generate new mutant lines as described previously^15^,^16^. The mutation was mapped by linkage analysis of offspring from a [(*stdf*/*stdf* x C3HeB/FeJ)F1 x *stdf*/*stdf* ] backcross using ABR thresholds at 8–10 weeks old to assign phenotype (n = 79 affected and 82 unaffected offspring). DNA from two distantly-related affected mice was submitted for exome sequencing using the Agilent SureSelect^XT^ mouse all exon kit for sequence capture, sequenced on the Illumina HiSeq 2000 platform and the data filtered and analysed as specified in Tables 1, 2, 3, 4, 5. The sequences have been deposited at ENA, accession number ERP000739. Mutant mice are available through EMMA.

### Modelling the mutation

S1pr2 was subjected to protein structure prediction using Phyre2 (Protein Homology/analogY Recognition Engine version 2.0^23^). The coordinates corresponding to the top hit, which was based on the X-Ray crystal structure of homologous receptor, S1pr1^24^, were used to produce a model of S1pr2 and to introduce the T289R mutation using Pymol™ (DeLano Scientific LLC).

### Immunohistochemistry

Inner ears of wildtype mice from the C57BL/6J strain carrying an albino mutation (C57BL/6Brd*Tyr*^*c-Brd*^) at postnatal day five (P5) and *S1pr2*^*stdf*/*stdf*^ mutants (with littermate *S1pr2*^+/*stdf*^ controls) at P14 and P28 were fixed and processed for immunohistochemistry. Sagittal wax sections, 8 μm thick, were exposed to rabbit anti-S1pr2 (21180-1-AP; Proteintech, Chicago, USA, 1:25), or anti-KcnJ10 (APC-035, Alomone Labs, Jerusalem, 1:300) antibody, before undergoing a DAB reaction and counterstaining with hematoxylin on a Ventana Discovery machine. Sections covering the entire inner ear for three different mouse samples of each genotype/age were examined.

### Electrophysiology

Auditory sensitivity was assessed using measurements of Auditory Brainstem Responses (ABRs)^17^. ABRs were recorded from Ketamine/Xylazine anaesthetised mice aged 4, 8 and 14 weeks, evoked by clicks and tone pip (6–30 kHz) stimuli presented freefield at 0–95 dB SPL in 5 dB steps to determine threshold sensitivity. EPs were measured under urethane anaesthesia (2 mg/g) in a different group of mice, in some cases following ABR threshold measurement. After these ABR recordings, a tracheal cannula was inserted and the bulla was opened to allow fenestration of the cochlear wall and insertion of a 150 mM KCl-filled glass micropipette into the scala media. The EP was measured as the potential difference between the scala media and a reference silver/silver chloride pellet under the dorsal skin of the neck^39^.

### Stria vascularis analysis

Inner ears were fixed in 4% paraformaldehyde at room temperature for 2 hours before the lateral walls were dissected out in PBS. Samples for cell boundary analysis were incubated with 488 phalloidin (1:400, Molecular Probes) in PBS at room temperature for 2 hours (P14, *S1pr2*^+/*stdf*^ n = 4; *S1pr2*^*stdf*/*stdf*^ n = 3; P28, *S1pr2*^+/*stdf*^ n = 7; *S1pr2*^*stdf*/*stdf*^ n = 7; P56, *S1pr2*^+/*stdf*^ n = 6; L, *S1pr2*^*stdf*/*stdf*^ n = 6). For capillary analysis, whole mount samples were isolated and incubated with isolectin GS-IB4, 594 (Molecular Probes, 1:50) at 4 °C, overnight in PBS with 3% BSA, to visualise capillaries (P14, *S1pr2*^+/*stdf*^ , n = 4; *S1pr2*^*stdf*/*stdf*^, n = 3; P28, *S1pr2*^+/*stdf*^ , n = 4; *S1pr2*^*stdf*/*stdf*^, n = 5; P56, *S1pr2*^+/*stdf*^ , n = 4; *S1pr2*^*stdf*/*stdf*^, n = 5). The samples were mounted with Vectashield Mounting medium with DAPI (Vector, Cat. No: H-1200) and imaged by confocal microscopy (Zeiss LSM 510 & 710). The numbers of capillary branch points per field (200 × 200 μm fields) in the middle turn of the cochlear duct was quantified using image J (controls, n = 4; homozygotes, n = 5, at P28).

### Scanning electron microscopy

Samples were taken from *S1pr2*^+/*stdf*^ and *S1pr2*^*stdf*/*stdf*^ mice aged 5 and 9 weeks old, a week after ABR testing at 4 and 8 weeks old respectively, and processed for scanning electron microscopy of the apical surface of the organ of Corti using the OTOTO methodology^40^. Samples were analysed according to their percentage distance from the cochlear base.

### Human population analysis

The 1958 British Birth Cohort and the collection of hearing data and analysis have been described previously^41^. Participants were drawn up from 17,638 individuals born in England, Scotland, and Wales in 1 week of March 1958. Of the original cohort, 9377 members were revisited by a research nurse for a biomedical follow-up in 2002–2004. Hearing measure consisted of pure tone audiometry at 1 kHz and 4 kHz at age 44–45 years and were adjusted for sex, nuisance variables (noise at test, nurse performing test, audiometer used in test), conductive loss, and hearing loss in childhood. DNA was collected from 6099 individuals and genotyped on various Illumina and Affymetrix SNP chips (for detail see http://www2.le.ac.uk/projects/birthcohort/1958bc/available-resources/genetic). These data were then imputed to the 1000 Genomes haplotypes (release March 2012) using MACH and Minimac. Measured SNPs with >95% call rate and Hardy–Weinberg p-value >0.0001 were included as input set. In subsequent analysis imputed SNPs with low imputation quality (r2-hat < 0.3 or MAF < 1%) were omitted. Individual associations were performed to hearing thresholds at 1 kHz and 4 kHz.

## Additional Information

**How to cite this article**: Ingham, N. J. *et al.* *S1PR2* variants associated with auditory function in humans and endocochlear potential decline in mouse. *Sci. Rep.* **6**, 28964; doi: 10.1038/srep28964 (2016).

## Figures and Tables

**Table 1:** Details of Exome Sequencing.

Mouse ID:	SD8.2c	SD23.1 g
Type of sequencing	Paired end	Paired end
Read length	79 bp	79 bp
Number of reads mapped	92726592	106900816
Mean depth	120.88x	139.48x
Coverage of bases in Agilent exons	99.88%	99.89%
Coverage of bases in Agilent exons to a depth of 10 fold or more	98.88%	99.11%
Coverage of bases in Agilent exons to a depth of 20 fold or more	96.24%	97.10%

**Table 2:** Details of exome analysis.

Analysis:	Software	Reference
Mapping to reference sequence (NCBIm37)	bwa 0.5.9	42
Local realignment around insertions and deletions	GATK 1.1–5	43,44
Lane merging and marking of duplicate fragments	picard 1.47	45, http://picard.sourceforge.net
Single nucleotide variant identification	SAMtools 0.1.17	46
Small structural variant identification	Pindel 0.2.4d	47

Steps of exome sequencing analysis and the software used at each stage.

**Table 3:** Filtered indels identified by Pindel and the primers used to check each by capillary sequencing.

Gene	Col5a3	Slc44a2	Rgl3	n/a
Position	9:20607549–20607551	9:21149853–21149855	9:21786028–21786030	9:24346521–24346523
Type	Deletion	Deletion	Deletion	Deletion
Size	1 bp	1 bp	1 bp	1 bp
Genetic location	intronic	intronic	intronic	Intergenic
Capillary sequencing results	Deletion not confirmed	Deletion not confirmed	Deletion not confirmed	Deletion not confirmed
In ancestral ES cell line	No	Yes	Yes	No
F primer	CATAGCTGGTTTGTGCATGG	GCGCAAAAGGATATTGATCG	AGAAGAGCTCCTGGGTAGGG	CCACATGTTCTGGACTTTGC
R primer	AGAGCCTGCGACAGTAGAGC	AGGTCACCAGTGGGTAGAGC	CAGAGCTCCTGGACTTCAGC	CCTCTGAAGGTTTGGAAAGG

Details of the four variations identified by Pindel which are present in both homozygous mice, have above average quality scores and are within the critical region, and the primers used to test each by capillary sequencing.

**Table 4:** Filtering steps applied to SNVs called by SAMtools.

Processing steps	Number of DNA changes
Input files	695728/775792
Mapping quality ≥45 and read depth ≥10	3200/3466
Present in both homozygotes	1556
Not present in dbSNP128 or the 17 wildtype strains	1164
On chromosome 9	46
Within mapped region	7
Exonic	3
Nonsynonymous	2
Not present in ES cells	1

SNVs were filtered first by quality and then by presence or absence in affected and wildtype mice, then by genomic location, predicted effect and presence in ancestral ES cells. The filter is described in the left-hand column and the number of SNVs remaining after each filter step is shown on the right.

**Table 5:** Filtered SNVs identified in the critical region on chromosome 9.

Gene name	Taf1d	Fat3	Zfp426	Col5a3	Rdh8	S1pr2	Bmper
Position	9:15116608	9:15834189	9:20280762	9:20602750	9:20627812	9:20772109	9:23211238
Reference	T	G	CGTGTGTGTGTGTGTGTGTGTGTGTGTGT	AACACACACACACACACACACACACACACACACACACACACAC	C	G	A
StoneDeaf	C	T	CGTGTGTGTGTGTGTGTGTGTGTGTGT	AACACACACACACACACACACACACACACACACACACACAC	T	C	C
Type	SNV	SNV	2bp deletion	2bp deletion	SNV	SNV	SNV
Location	Intronic	Intronic	Intronic	Intronic	Exonic	Exonic	Exonic
Zygosity	Homozygote	Heterozygote	Heterozygote	Heterozygote	Homozygote	Homozygote	Heterozygote
Consequence (if in coding region)					synonymous	T289R	T570P
In ancestral ES cell line	Yes	No	No	Yes	No	No	Yes

Details of the SNVs which passed the first five filtering steps in Table 4. They had a high mapping quality and read depth, were present in both mice sequenced, were not present in any wildtype strains or dbSNP128, and were within the mapped region.
